# Finite element analysis and clinical study of chest wall reconstruction using carbon fiber artificial rib

**DOI:** 10.1038/s41598-023-50716-x

**Published:** 2024-01-02

**Authors:** Xiang Zhang, Zhixia Cai, Bo Liu, Jiqiao Liao, Fenglei Yu, Zhoujian Tan, Bin Wang, Mei Yang, Bowen Zhang

**Affiliations:** 1 Hunan Tankang Biotech Co., LTD., Changsha, 410083 Hunan Province People’s Republic of China; 2 Department of Thoracic Surgery, the Second Xiangya Hospital, Central South University, Changsha, 410011 Hunan Province People’s Republic of China; 3 College of Mechanical and Electrical Engineering, Central South University, Changsha, 410083 Hunan Province People’s Republic of China

**Keywords:** Implants, Biomedical materials

## Abstract

Carbon fiber composites are emerging as a promising new biomaterial for chest wall reconstruction implants due to their mechanical properties and biocompatibility. This work evaluates the biomechanics of carbon fiber artificial ribs using finite element analysis and clinical implementation. Static simulations of normal breathing process show the maximum stress on the implant is only 2.83 MPa, far below the material ultimate strength of 60 MPa, indicating the excellent fit for maintaining respiratory function. Dynamic collision simulations demonstrate the artificial rib model could withstand a 4 kg rigid object impact at 2 m/s without fracture. Reconstructing the artificial rib with a human rib in the finite element analysis model increases the overall stress tolerance. The impact force required for fracture increases 48% compared to the artificial rib alone, suggesting improved strength from rib integration. Clinically, 10 of 13 patients receiving the artificial rib implants show no significant loss of pulmonary function based on spirometry tests. Based on our findings, the combined simulations and clinical results validate the strong mechanical performance and biocompatibility of the carbon fiber artificial ribs for chest wall reconstruction under static and dynamic loading while maintaining normal respiratory function.

## Introduction

Chest wall defects can arise from various chest wall diseases, including tumors, congenital malformations, or complex infections. Surgical resection of these lesions often necessitates the removal of the affected chest ribs, resulting in local skin, muscle, and bone deficits^1,2^. Failure to promptly complete bony reconstruction of the chest wall to restore its integrity and stability can lead to chest wall weakening, abnormal breathing, exacerbated respiratory problems, and circulatory disorders^3^. Therefore, chest wall reconstruction stands as the most effective clinical approach for addressing substantial chest wall defects. Several key considerations should generally be taken into account for chest wall reconstruction^4^: (1) Chest wall repair materials must possess adequate rigidity to safeguard the chest and upper abdominal organs. (2) The integrity of respiratory function should be maintained after chest wall reconstruction. (3) The reconstructed thorax should enable full range of motion and strength in the upper limbs and shoulder joints. (4) Chest wall reconstruction materials should exhibit high safety, implantability, non-carcinogenic properties, promote fibrous tissue growth, and resist infection. (5) It should not interfere with chest X-ray examinations and allow for convenient patient follow-up.

At present, the commonly used chest wall reconstruction materials in clinic include titanium alloy materials (such as Matrix-RIB system, Stratos system), synthetic materials (such as methyl methacrylate, polytetrafluoroethylene, and polypropylene) or biological bones (autologous rib transplantation or biological rib). These traditional chest wall reconstruction materials often fall short of meeting the actual clinical requirements. For instance, metal materials have a notably high elastic modulus, leading to a pronounced stress shielding effect. This frequently results in clinical complications post-implantation, such as infections, pain, and the risk of chest viscera puncture. On the other hand, synthetic and patch materials lack the ability to provide the essential stability and integrity required for a fully functional thorax. Their relative softness hinders them from adequately protecting visceral organs and maintaining normal respiratory and circulatory functions. Furthermore, biological rib and similar materials face limitations in terms of scalability, making it challenging to achieve mass production and effectively repair extensive damage^5,6^. Consequently, there is an urgent need to develop new bone-based chest wall reconstruction materials for clinical use.

Carbon/carbon (C/C) composite is a promising biomaterial characterized by carbon fiber reinforcement and a pyrolytic carbon matrix. Its exceptional properties, including low density, an elastic modulus that aligns with human bone, excellent biocompatibility, and the absence of artifacts in medical testing (NMR and CT scan), have led to its extensive application in clinical research involving bone plates, dental roots, heart valves, blood vessels, tendons, and various other tissues^7–9^. In the realm of medical innovation, artificial ribs constructed from C/C composite represent a significant stride towards the development of safe and effective solutions for chest wall reconstruction surgery. For example, Wang et al. implanted uncoated C/C composite into New Zealand white rabbits to observe the adhesion and combination of fibrous tissue on C/C composite^10^. Su et al.^11^ used three-dimensional finite element analysis method and computer simulation to analyze the stress and strain state of C/C artificial femur, which provided theoretical and design basis for C/C molding process. Zhang et al.^12^ compared the acute biocompatibility between carbon fiber composite artificial skull and titanium mesh (skull implant used in clinic at present). They show that the biological performance of carbon fiber composite artificial skull is better than titanium mesh. However, there are few reports regarding the application of C/C materials in artificial ribs, especially on chest wall reconstruction.

On the other hand, the structure and composition of human bones are complex, and conducting experiments to examine the mechanics of actual human bones presents significant challenges and limitations. This complexity has hindered comprehensive studies of human bone. However, the advancement of finite element method has played a pivotal role in facilitating research of the structure of human bones. For instance, Torcasio et al.^13^ have effectively utilized finite element analysis based on Micro CT to accurately assess bone strain near tibial implants in rat models. Lughmani et al.^14^ have reached excellent agreement between average critical thrust and torque values in cortical bone during bone plastic surgery, as derived from finite element analysis, and experimental results. Similarly, Bustillos et al.^15^ have demonstrated that finite element simulations of femur failure load align closely with actual mechanical test results. Moreover, Li et al.^16^ have examined the sensitivity of mesh density, cortical thickness, and material properties in rib through finite element models, providing valuable insights for creating chest models in impact biomechanics. Johan et al.^17^ have developed a comprehensive human rib model in finite element method, which enabled the prediction of human rib fracture risk in vehicle collisions. This application of finite element analysis allows for the accurate evaluation of stress on human bones, offering valuable guidance for the selection of materials and structural design in the development of artificial bones.

In view of above conditions, this paper focuses on carbon fiber composite material as an artificial rib material, conducting a comprehensive analysis of its biomechanical properties. Through finite element analysis, we investigate the behavior of the entire artificial rib model, single artificial rib model, and their interaction with single human ribs under static and dynamic stress conditions. The vulnerable areas of the entire thoracic region are evaluated to provide a more thorough understanding of thoracic damage across different conditions. In addition, we delve into the response of individual ribs by establishing a dedicated single rib model. Finally, in-depth analysis among the complete artificial bone, single artificial rib, and human bone reconstruction is reached. The biomechanical properties of carbon fiber composite artificial ribs explored in this work would offer valuable insights into their clinical utility, particularly in the context of chest injuries frequently encountered in daily life.

## Finite element model (FEM)

### CT image acquisition

The original model of the human chest and ribs was obtained by CT image acquisition. The object of CT image acquisition was a 70-year-old male patient, 160 cm tall and 40 kg in weight, who was diagnosed with multiple myeloma. The CT scanner for Siemens Somotom Force Dual Source was made by Siemens. The scanning condition is 100 kV, and the CT image is reconstructed into a 1 mm thick CT image, 324 slices are used during model construction. The resolution of each slice is 512 * 512 pixel, and a spacing of 22 is used in each pixel. CT images are stored as medical digital imaging and communication (DICOM) format files and output to the medical 3D reconstruction software, Mimics 21.0.

The construction of a CAD model is as follows. Firstly, the Hounsfield Unit value larger than 1000 is used to select the cortical bone used in the chest wall reconstruction. Secondly, the cortical bone model from CT images in Mimics 21.0 software outputs as an STL format file, and is imported into Geomagic Wrap 2017 software. The original STL file is optimized in Geomagic Wrap 2017, such as feature removal, noise reduction, meshing, and surface fitting processing. After optimization, a three-dimensional model is built for the chest and ribs, which is output into another STL file. Thirdly, the optimized STL file is input into the Solidworks 2017 software and reconstructed the chest and rib model of the composite human structure through boolean operations. Finally, a STEP file is output after processing in Solidworks 2017 software.

### Establishment of integral artificial rib model

The 1:1 scanning of the real human chest bone model introduces irregular shapes in both the sternum and ribs, with surfaces comprised of diverse curved elements. This intricacy presents a challenge in establishing meshing and boundary conditions for static and dynamic simulations of artificial ribs using this model.

**Figure 1:**
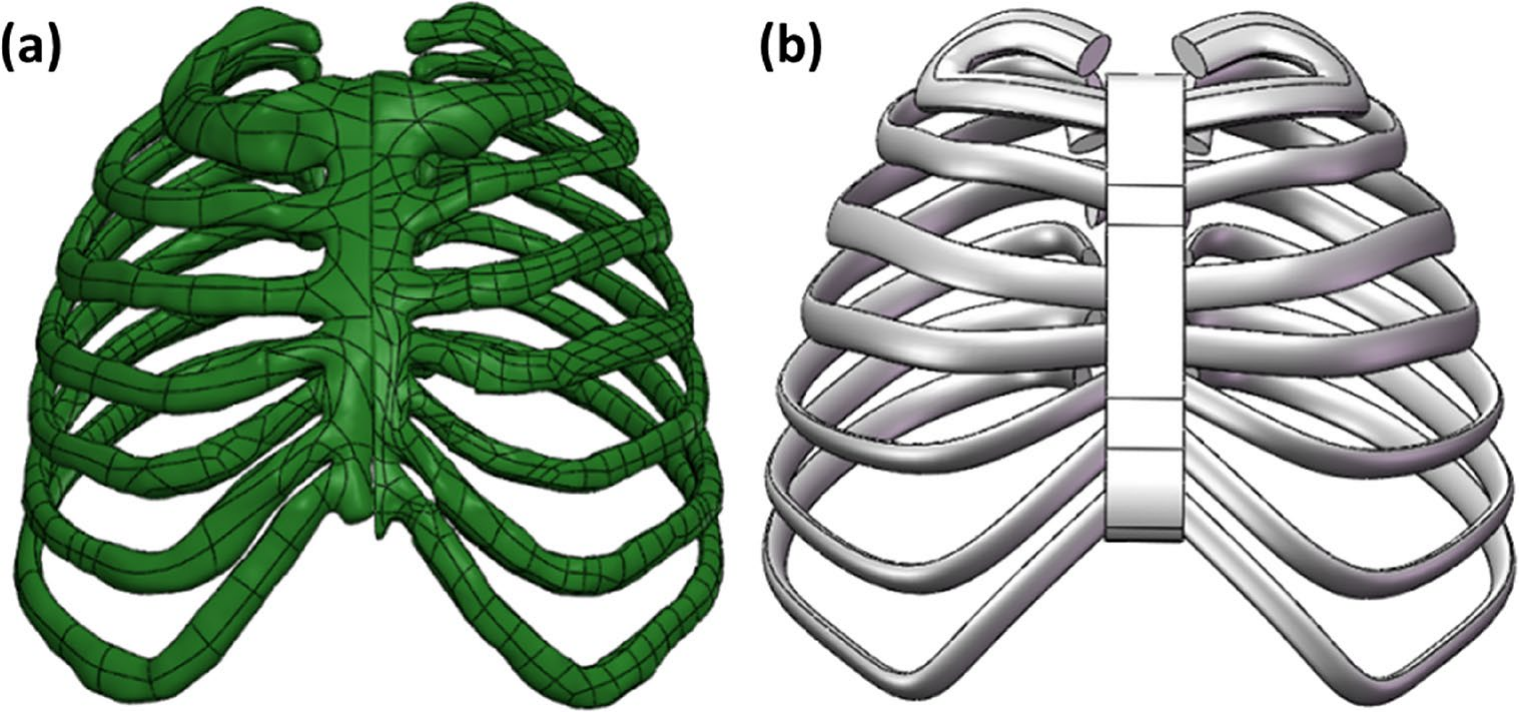
Comparison of artificial bone models before and after simplification, (**a**) original artificial bone model; (**b**) simplified model of artificial bone.

To address this issue, we utilized the three-dimensional model of the original scanned artificial bone. Here, we simplified the rib cross-section to resemble an ellipse, akin to a CT cross-section. Additionally, we approximated the side contour of the sternum using a spline curve. By strategically selecting coordinate points on the rib section of the original model, we derived the contour curve for the rib section through fitting. Subsequently, we reconstructed the rib section along this contour curve utilizing SolidWorks' scanning function. The outcomes before and after this simplification process are illustrated in Fig. 1.

While the overall structure of the simplified model closely mirrors that of the original, this simplification significantly streamlines the meshing process and the application of load boundary conditions in subsequent static and dynamic simulations. Consequently, this leads to an improvement in the efficiency of the simulation calculations.

### Establishment of a single artificial rib model

**Figure 2:**
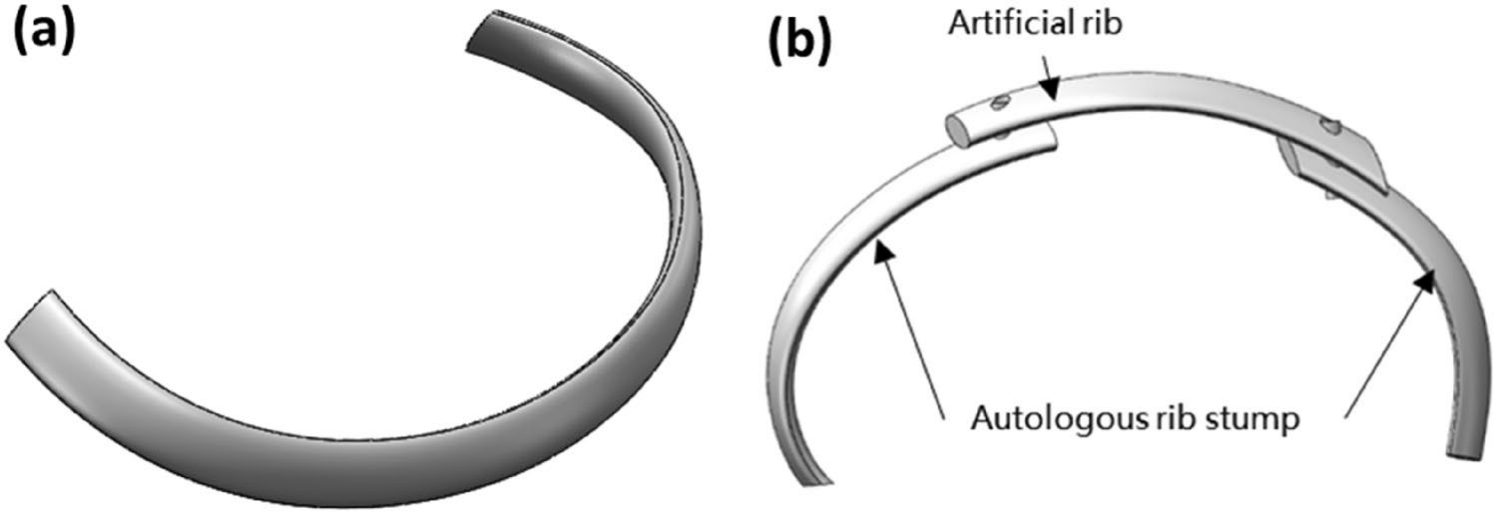
Single rib model, (**a**) artificial rib; (**b**) artificial rib and human bone lap.

Our approach to analyzing artificial bone follows a structured progression from the whole to the specific components. After creating the overall model, we employ a similar methodology to model individual ribs, as depicted in Fig. 2a. This step-by-step process allows for a more detailed examination of the local aspects of the model, enhancing the accuracy and precision of our analysis.

### Establishment of artificial rib and human bone reconstruction model

In scenarios where the human body sustains damage from collisions or other injuries, and some ribs cannot be recovered, artificial bones come into play to replace the damaged ribs. This restoration process aims to reinstate normal thoracic function. The diagram in Fig. 2b illustrates the model depicting the overlapping of artificial ribs and human bone following the repair of autologous ribs with artificial bone. It’s important to note that the schematic model differs from the actual application of artificial ribs in terms of the connection method. In practical use, artificial ribs and autologous ribs are typically connected using medical sutures or stainless-steel wire binding. However, due to the simplification of our simulation model, achieving a precise surface fit between the artificial bone and the autologous rib becomes challenging, making it difficult to accurately simulate the effect of medical suture binding. Consequently, in our simulation model, we employ two bolts to establish the lap connection. During the simulation process, we ensure that the artificial bone and autologous rib become fully bonded through the application of external force, effectively replicating the real-world effect of the connection.

### Artificial bone material properties

**Figure 3:**
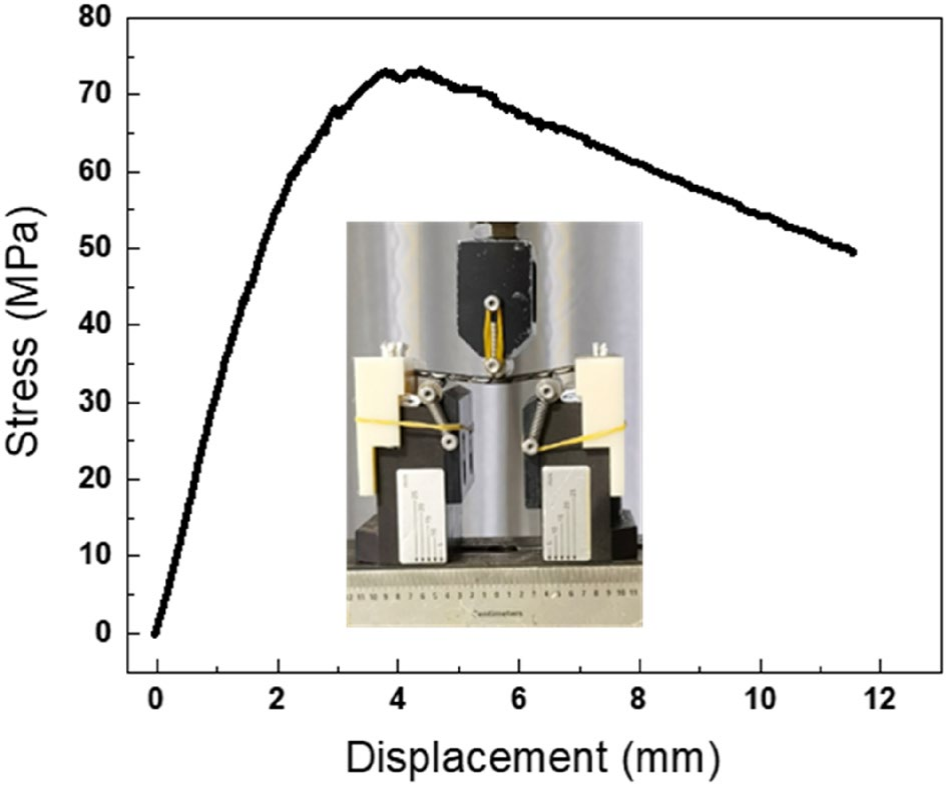
Displacement-stress diagram of carbon fiber artificial rib.

The raw material selected for the artificial bone is Carbon fiber PAN-T700-12k from Zhongfu Shenying Carbon Fiber Co., Ltd. This material undergoes a process of re-braiding and shaping, followed by preparation through chemical vapor deposition, using natural gas as the carbon source. The corresponding assembled module has material properties far below that of pristine carbon fiber materials. As shown in Fig. 3, the mechanical analysis of the resultant carbon fiber artificial rib reveals a stress-deformation behavior divided into two distinct stages. Initially, in the elastic deformation stage, strain increases linearly with external force. Upon removing the external force, the artificial bone returns to its original state. Subsequently, in the plastic deformation stage, internal stress exceeds the yield stress. Even after the external force is removed, the artificial bone remains partially deformed and cannot fully revert to its original state. With continued application of force, the artificial bone eventually reaches its strength limit, leading to fracture. Noted this nonlinear elastic–plastic behavior when subjected to damage is well documented, as shown in the stress-displacement curve of Fig. 3. However, the maximum stress experienced by chest wall is far below the yield stress of carbon-fiber materials. Thus, it is safe to assume a linear (near elastic) behavior of carbon-fiber materials. That is why we used the constant material parameters of artificial bone listed in Table 1. These include parameters such as elastic modulus, Poisson's ratio, yield stress, and strength limit. The specific material parameters for the carbon fiber artificial rib model are detailed in Table 1.

**Table 1:** Material parameters of artificial bone.

Density (g/cm^3^)	Modulus of elasticity (GPa)	Poisson’s ratio	Yield stress (MPa)	Strength limit (MPa)	Yield strain	Maximum strain
1.5	4	0.3	40	60	0.014	0.025

### Mesh size and method of FEM

**Figure 4:**
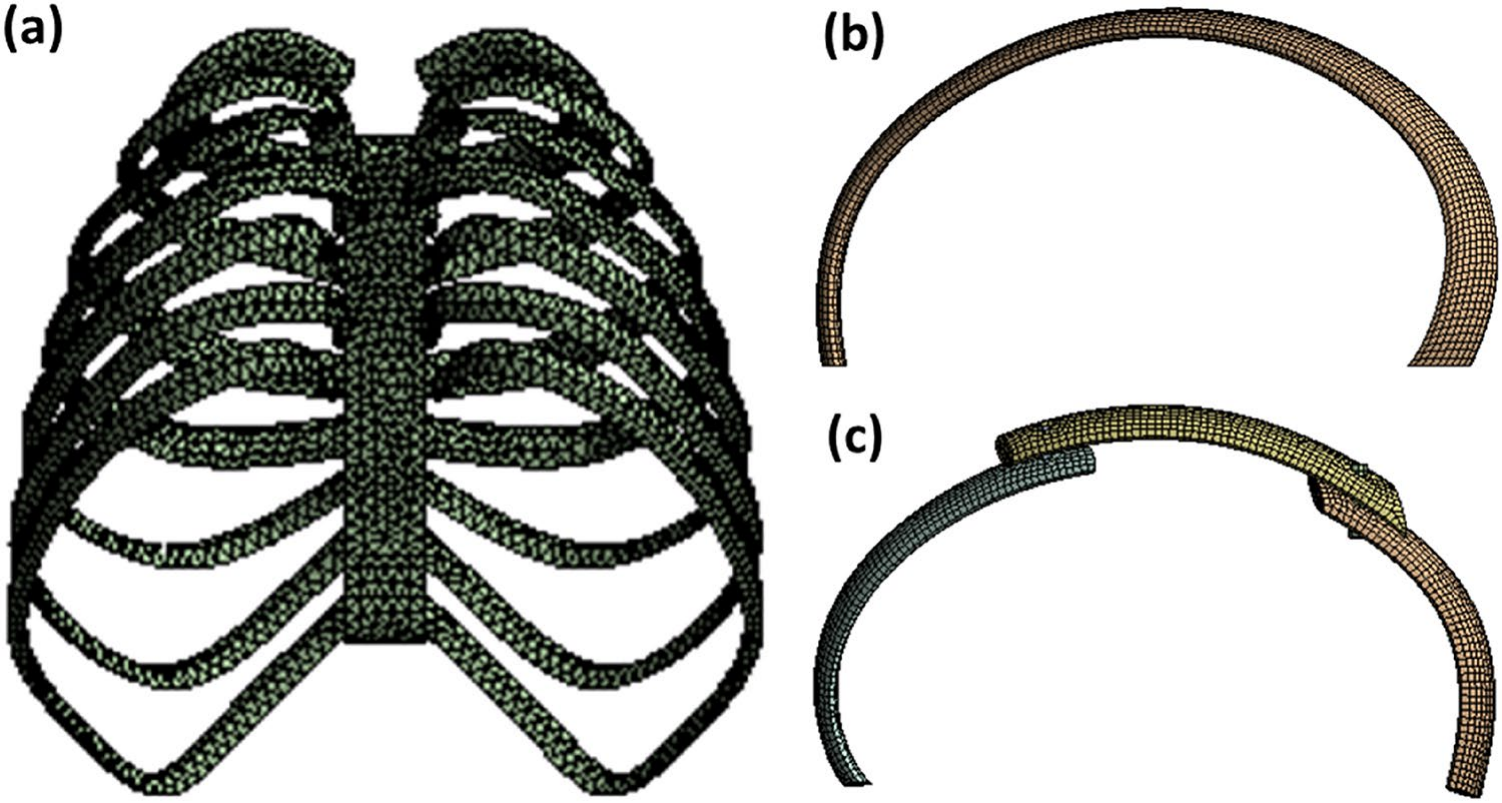
The mesh of FEM, (**a**) artificial bone; (**b**) single artificial rib; (**c**) reconstruction rib.

The mesh division method plays the key role in the accuracy of finite element model calculation. In the first place, the mesh size and mesh model have been tested in an ascending order and the converged one is chosen for large scale simulation. As a result, different meshing methods are used for artificial bone model, single rib model and reconstructed rib model. As listed in Table 2, a tetrahedral mesh with a grid size of 5 mm is used to speed up the simulation of artificial bone. A hex-dominant mesh method with a grid size of 2 mm is used for the single rib model and reconstruction rib. The typical meshing results are shown in Fig. 4 and the convergence is reached. The corresponding mesh size are listed in Table 2.

**Table 2:** The mesh result of FEM.

	Mesh method	Mesh size/mm	Mesh number	Nodes
Artificial bone	Tetrahedrons	5	27,517	13,122
Single artificial rib	Hex-dominant	2	11,168	14,003
Reconstruction rib	Hex-dominant	2	14,289	15,444

### Loading and constraints

Statics primarily focuses on the state of artificial ribs during normal breathing, where individuals inhale and exhale. During inhalation, the chest expands, the diaphragm descends, the pleural cavity’s volume increases, and chest pressure decreases, allowing oxygen to be drawn into the lungs from the atmosphere. At the end of inhalation, chest pressure typically ranges from − 8 to − 4 cmH_2_O. Exhalation reverses this process: the pleural cavity volume decreases, chest pressure rises, and carbon dioxide is expelled from the lungs. At the end of exhalation, chest pressure ranges from − 4 to − 2 cmH_2_O. In static analysis, we select the extreme case of maximum thoracic pressure for examination, specifically the scenario where the maximum pressure difference between the inside and outside of the thorax at the end of inspiration is − 8 cmH_2_O.

**Figure 5:**
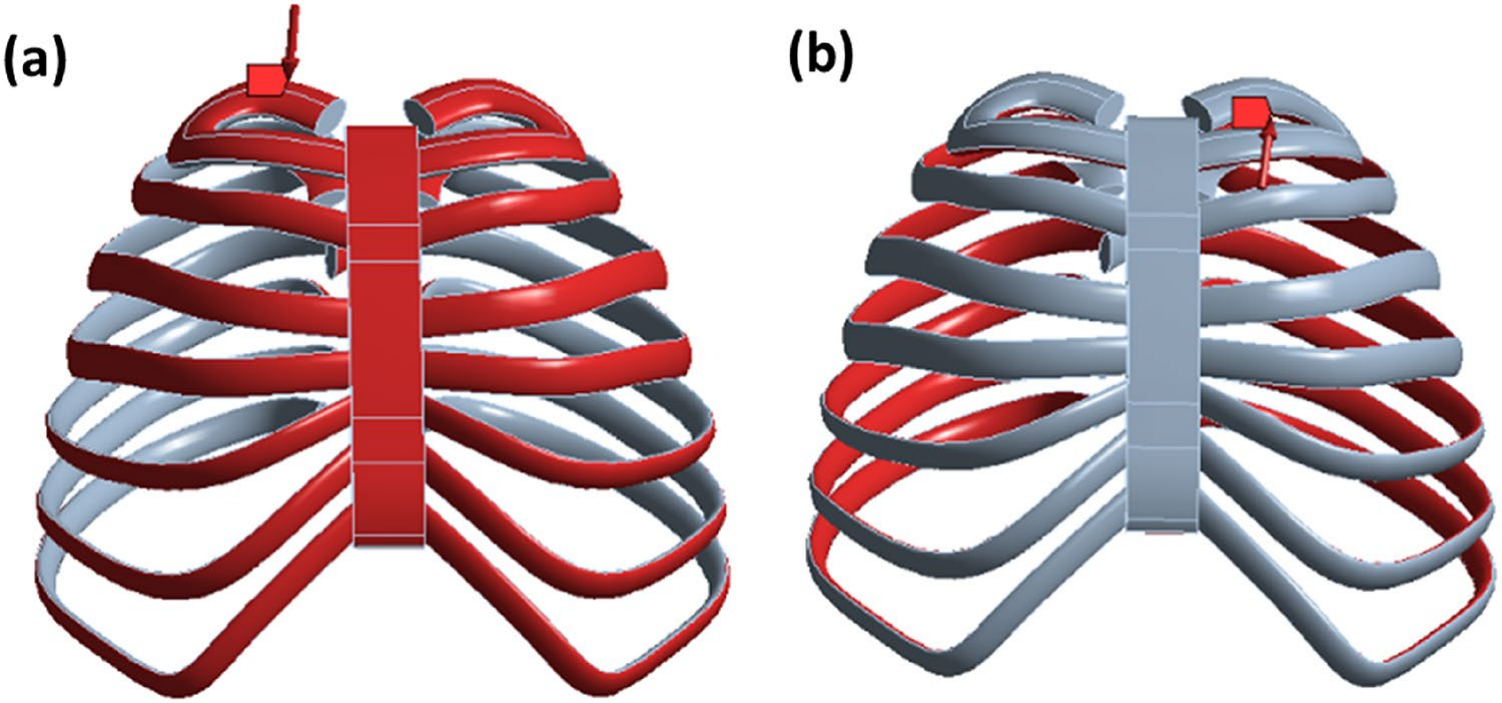
Artificial bone model loaded in statics, (**a**) Internal thoracic pressure; (**b**) External thoracic pressure.

We use this as the simulation condition and compare and analyze the whole artificial bone model, single rib model, and the artificial bone and rib junction model. In the statics analysis, the three models are loaded in a similar way, and the artificial bone model, for example, is loaded as shown in the Fig. 5.

**Figure 6:**
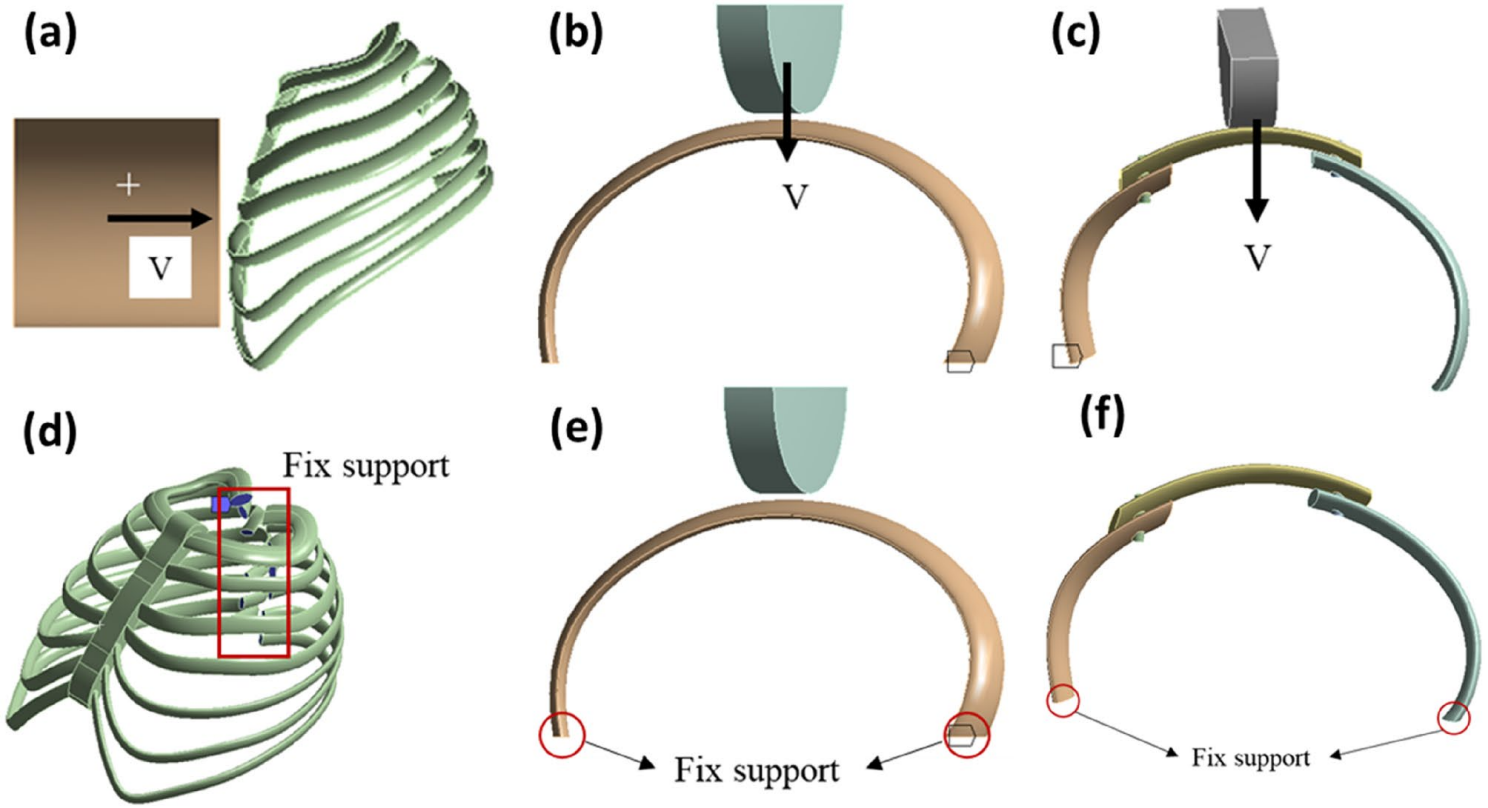
Different model, loads in dynamics, (**a**) artificial bone; (**b**) single artificial rib; (**c**) reconstruction tib, constraint settings, (**d**) artificial bone; (**e**) single artificial rib; (**f**) reconstruction rib.

Dynamics, on the other hand, centers on how artificial bone responds to dynamic forces, such as impacts. In dynamic analysis, we simulate frontal collisions with the entire model. The impacting mass is 4 kg, and the collision speed is 2 m/s. These values are chosen to capture the complete transformation process of artificial bone during collisions and assess the critical positions of artificial bone at different phases of the collision. For the local model (comprising the single rib model and the artificial rib incorporated into the human bone model), the impactor strikes the center of the model at a constant speed of 4 m/s to evaluate the load-bearing capacity of these relatively delicate structures. To expedite simulation calculations, we treat the impactor as a rigid body during the collision process. In dynamics, different models are loaded as shown in Fig. 6a–c. In the finite element model, the artificial bone model, the single rib model and the reconstructed rib model all set fixed constraints at the end of the ribs, as shown in Fig. 6d–f.

Regarding constraint settings, we simplify calculations by applying fixed constraints at the rib's end in both static and dynamic simulations. All the finite element analysis is performed on the ANSYS software.

### Clinical validation

The optimized artificial rib structure is adopted for clinical validation. Carbon fiber artificial ribs are used to perform chest wall reconstruction for 13 patients with chest wall defects. All research was performed in accordance with guidelines/regulations of the Second Xiangya Hospital at Central South University, and also in accordance with the Declaration of Helsinki. All procedures involving patients received approval from the Clinical Trial Ethics Committee of Central South University, People's Republic of China (No. SYXK 2017-0002). Written informed consent was obtained from the individual for participation in the study, and for the publication of any potentially identifiable images or data included in this article.

### Ethical approval

All procedures involving patients received approval from the Clinical Trial Ethics Committee of Central South University, People's Republic of China (No. SYXK 2017-0002). Written informed consent was obtained from the individual for participation in the study, and for the publication of any potentially identifiable images or data included in this article.

## Results and discussion

### Mechanical simulation results of artificial bone

#### Static analysis

**Figure 7:**
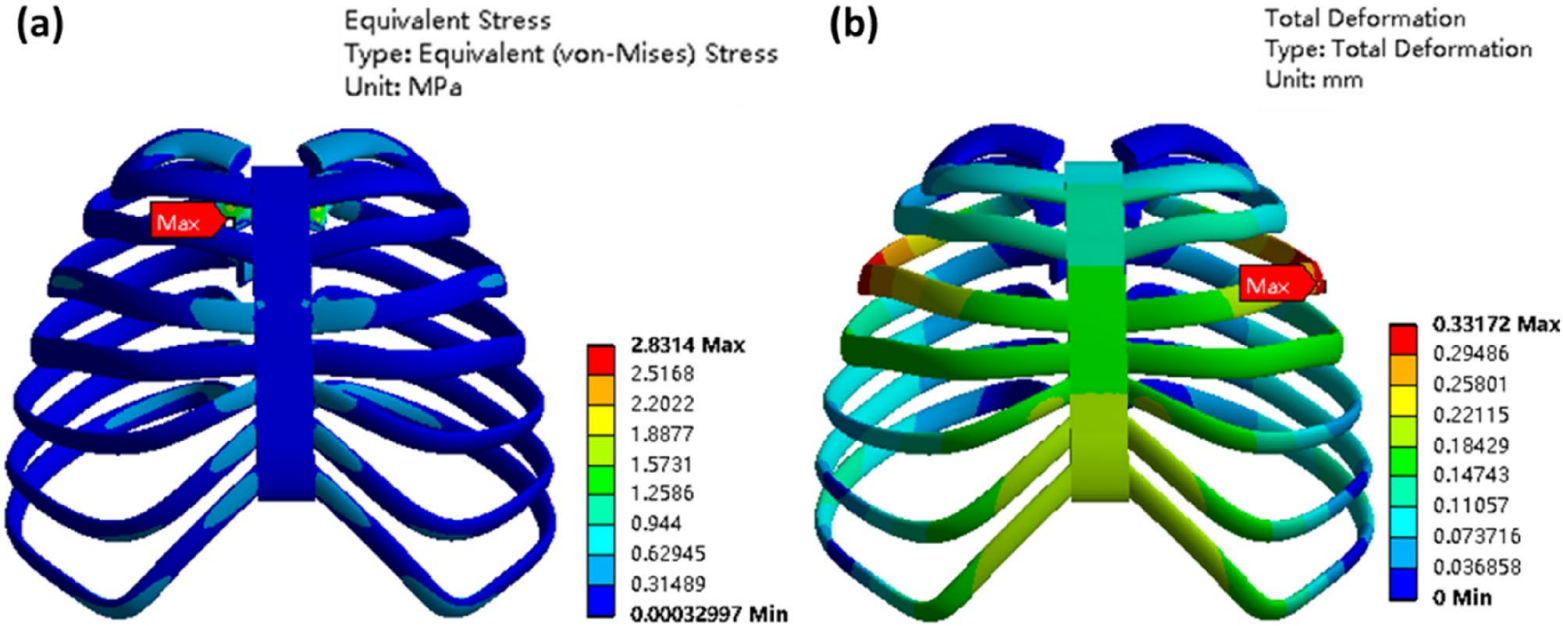
Model of integral artificial rib, (**a**) stress; (**b**) deformation.

Improper material usage in chest wall reconstruction surgery can decrease a patient’s vital capacity by 15%, significantly impacting post-surgical normal breathing^18^. Consequently, it is crucial to investigate the stress experienced by carbon fiber artificial ribs during normal breathing after implantation into the human body. Converting pressure units from cmH_2_O to Pa using the relationship 1 cmH_2_O = 97.8 Pa, we impose a pressure of 0.1005 MPa on the artificial bone model. The external pressure on the artificial bone model equals atmospheric pressure, which is 0.1013 MPa. Mesh quality analysis yields a value of 0.7758, exceeding the 0.7 threshold suitable for biomechanical finite element analysis in human studies. Stress–strain results for the final artificial bone model are depicted in Fig. 7.

Upon analyzing the stress results, we observe that at the end of normal inhalation, the artificial rib experiences minimal overall stress. Stress concentration occurs primarily at the front and end of the rib, with the highest stress occurring at the end of the third rib, reaching 2.83 MPa. This value differs little with other parts of the constructed chest wall, indicating good compatible after construction. Regarding strain results, artificial bone deformation is primarily concentrated in the middle and frontal sections of the model. The greatest deformation occurs in the middle of the third rib, measuring 0.332 mm.

#### Dynamics analysis

**Figure 8:**
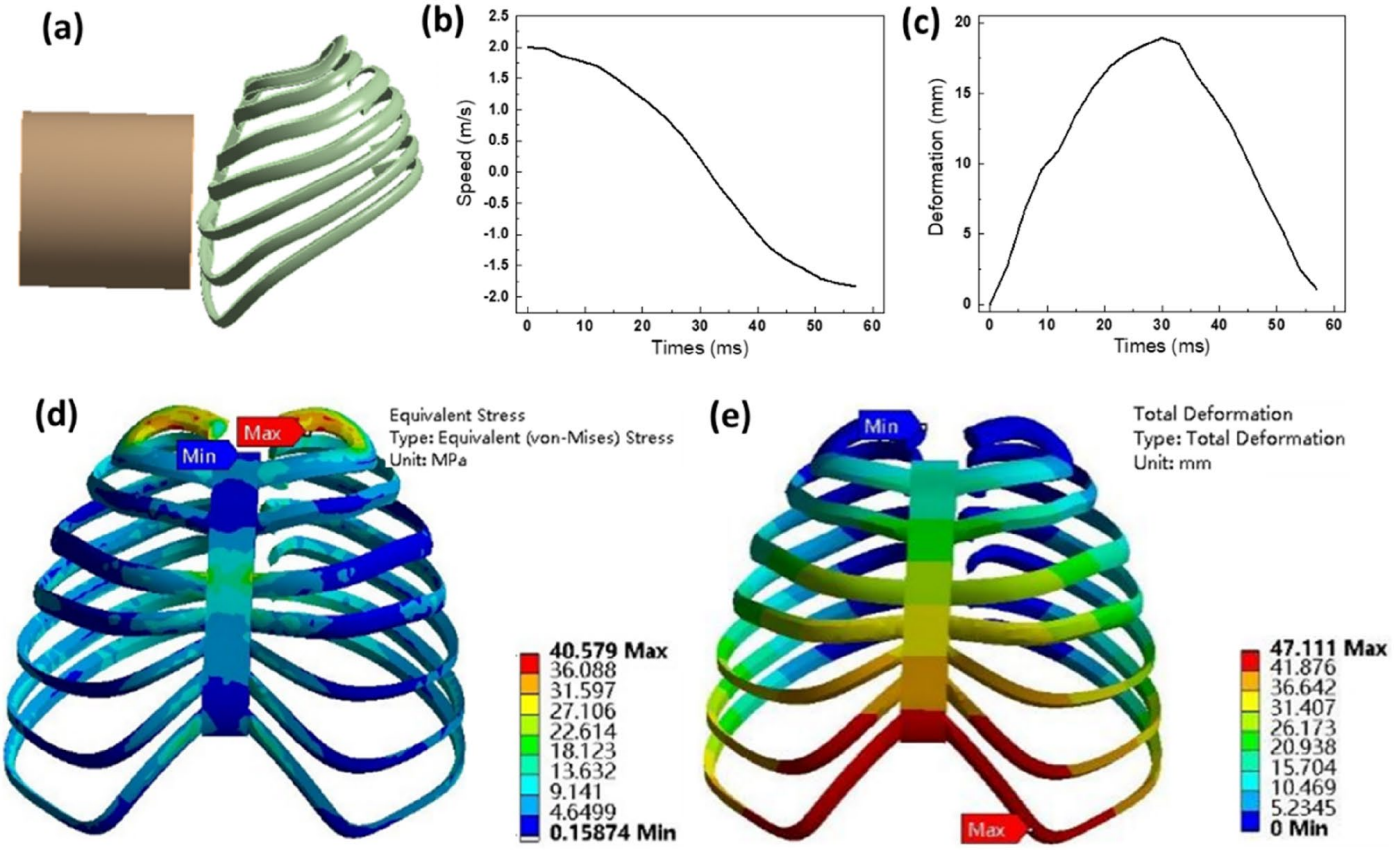
Frontal collision simulation results of artificial rib overall chest model, (**a**) frontal collision simulation model; (**b**) impactor speed change; (**c**) deformation time curve of artificial rib overall model; Over all chest at 33 ms, (**d**) stress; (**e**) deformation.

We employ a rigid impactor with a mass of 4 kg and an initial impact velocity of 2 m/s to collide with the artificial rib, as shown in in Fig. 8a. The velocity–time history curve resulting from impacting the chest's front is presented in Fig. 8b. This curve reveals that upon contact with the chest, the impactor experiences resistance, leading to a rapid drop in its velocity, reaching its minimum at 33 ms. This reduction in speed primarily stems from the deformation of the ribs, which absorb energy. At the point of minimum velocity, the thorax section undergoes a rebound, causing the impactor to regain a certain reverse speed.

To illustrate the artificial rib's deformation during the collision more effectively, we've plotted the curve depicting the average deformation of the artificial rib over time, as seen in Fig. 8c. Between 0 and 33 ms, the chest undergoes compression and deformation due to the impactor's force. From 33 to 60 ms, the chest experiences impact and binding, leading to a rebound effect. Throughout the entire impact process, the artificial rib reaches its maximum deformation at 33 ms.

Examining the artificial bone model at 33 ms, we assess its overall deformation and stress, as depicted in Fig. 8d,e. Our simulation results reveal a relatively low overall stress distribution in the artificial bone model, with the highest stress reaching 40.58 MPa at the rib's end. The most significant deformation measures 47.11 mm and occurs at the front of the final rib.

**Figure 9:**
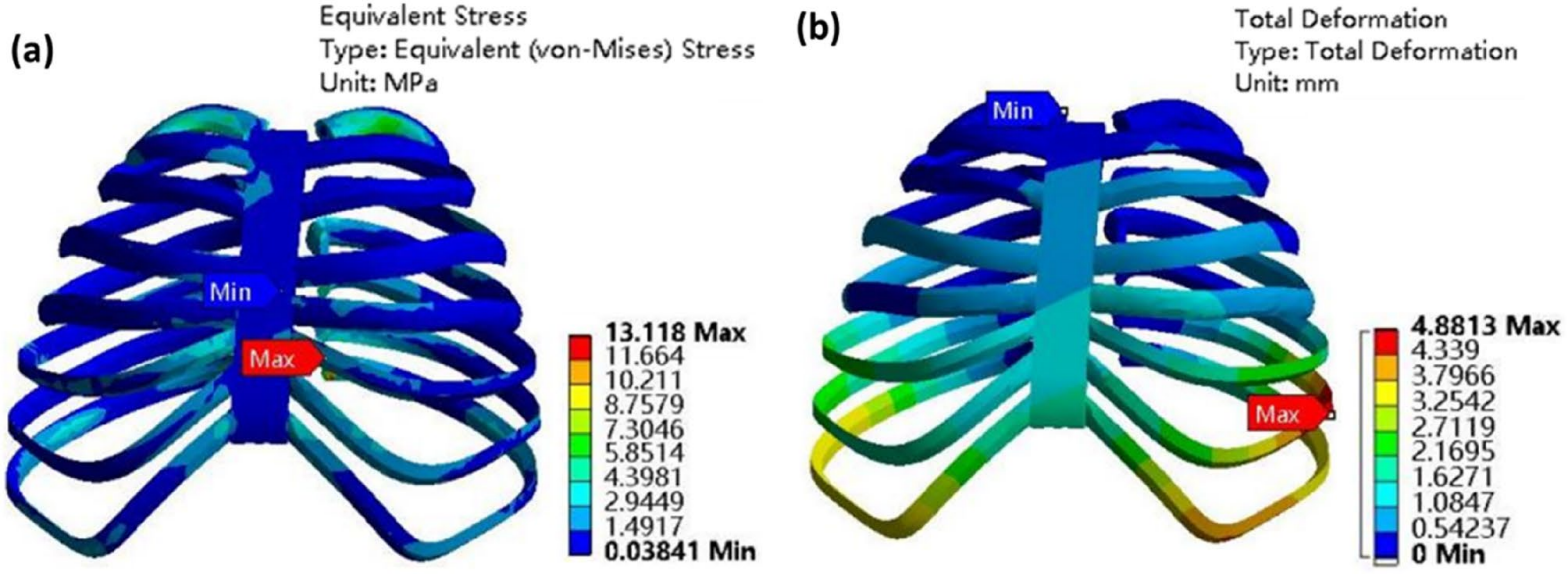
Simulation results of artificial bone model after impact, (**a**) overall chest stress; (**b**) overall chest deformation.

Figure 9 illustrates the overall deformation and stress in the artificial bone model following the impact. The maximum stress observed is 13.12 MPa, with the most significant deformation measuring 4.88 mm. The maximum stress locates at the back of the rib cage. This is due to the load passes to the back after impact. The front bounces back to its normal shape, while the back is subject to more loading. It's important to note that the current models exclusively encompass bones, lacking representations of human muscles, internal organs, or other tissues. Consequently, it is reasonable to infer that the deformation outcomes obtained from simulation may exceed the actual collision-induced deformations.

### Mechanical simulation of single rib model

#### Static analysis

**Figure 10:**
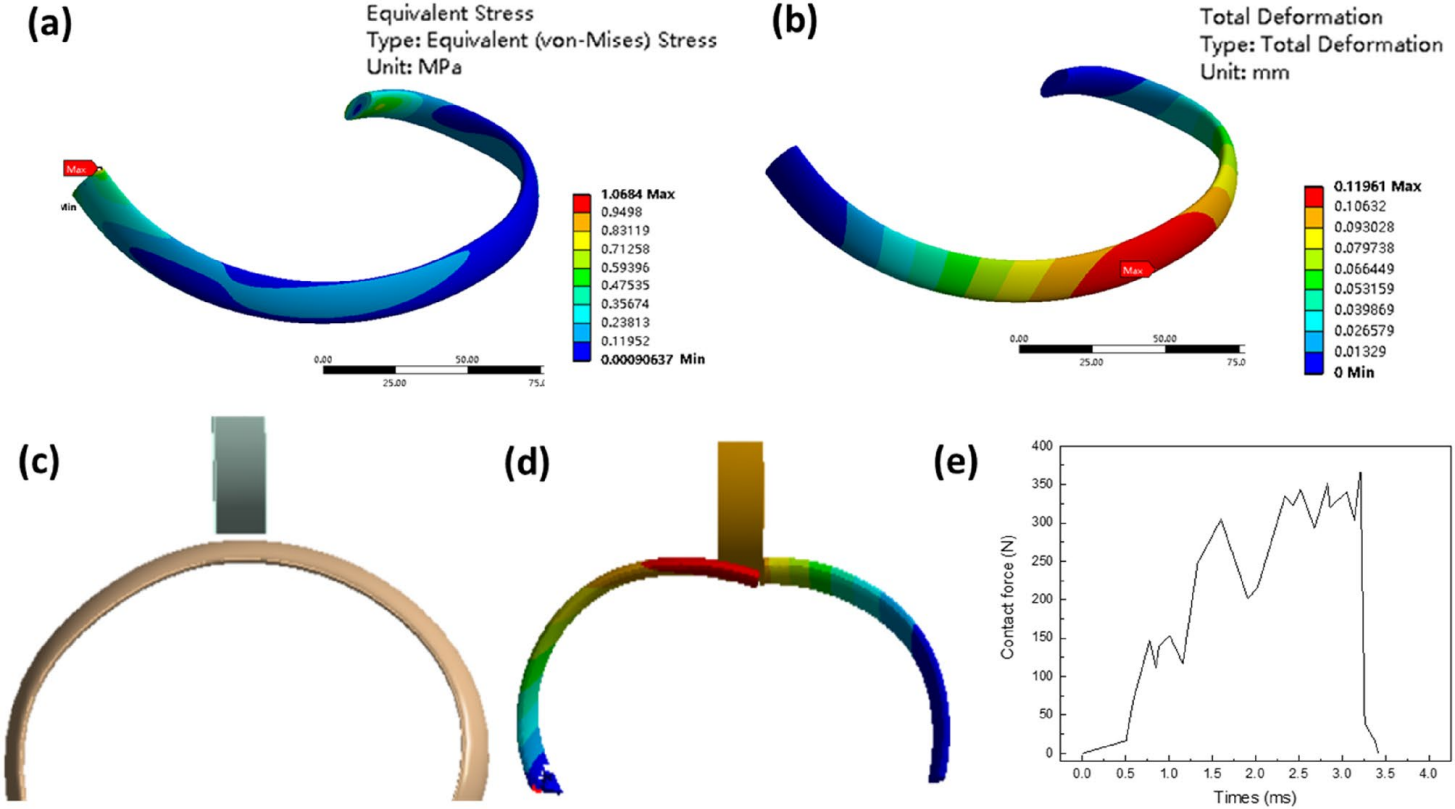
simulation results of single artificial rib model, static analysis, (**a**) single rib model stress; (**b**) single rib model deformation; frontal impact simulation, (**c**) model diagram; (**d**) rib fracture; (**e**) rib contact force time curve.

The method for imposing constraints and loads on a single artificial rib closely mirrors the approach used for the overall artificial bone model. However, when applying constraints to a single artificial rib, both ends of the rib are fixed and restrained, while the other procedures remain consistent with those used for the overall artificial bone model. The resultant stress and deformation outcomes for a single rib are presented in Fig. 10a,b. After subjecting a single rib to the same respiratory load as applied to the whole model, we observe a maximum stress of 1.07 MPa, concentrated at the rib's end. Furthermore, the maximum deformation, measuring 0.12 mm, is situated in the middle of the rib. These results align with the conclusions drawn from the analysis of the entire artificial rib model.

#### Dynamic analysis

In dynamic simulation, we directed the impact object to strike the center of a single rib at a specific velocity. Fixed constraints were applied to both ends of the rib, and the collision object was set to collide at a constant speed of 4 m/s, targeting the rib. The schematic representation of this setup is depicted in Fig. 10c–e. The graphs, Fig. 10e, display the contact force against time between the collision object and the rib. It is evident that the contact force experiences a gradual increase as the impact object strikes the single artificial rib. This force then experiences a rapid decline when it reaches its peak, signifying the point at which the artificial rib fractures. The maximum contact force recorded is 367 N.

### Mechanical simulation of reconstructed rib model

#### Static analysis

**Figure 11:**
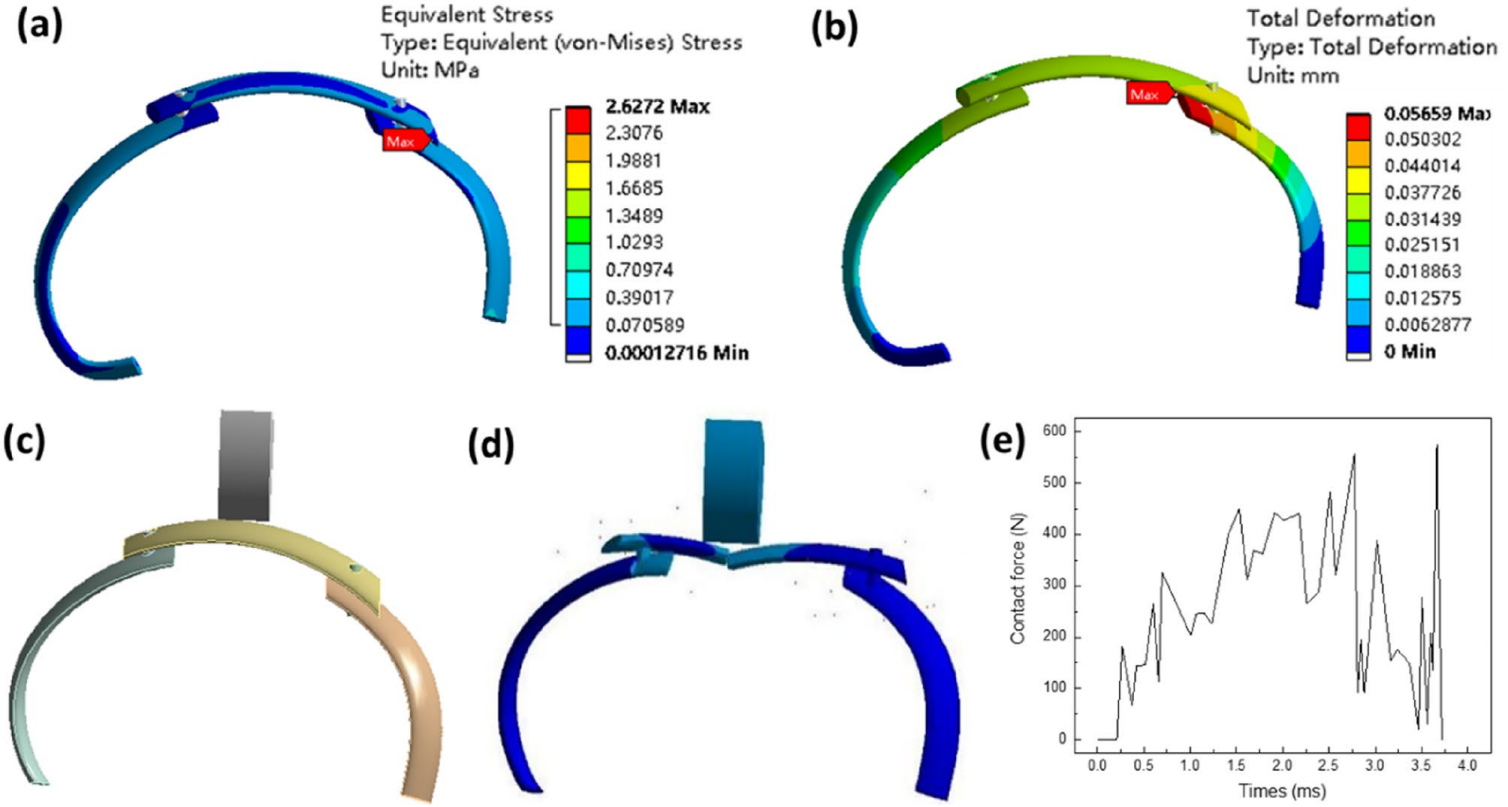
simulation results of rib reconstruction model, static analysis, (**a**) stress; (**b**) deformation; frontal impact simulation, (**c**) schematic diagram of model; (**d**) rib fracture; (**e**) contact force time curve.

To simulate the static analysis process of a single rib, we applied the same respiratory load and constraints to the rib reconstruction model as in the single rib scenario. The obtained stress and deformation results are depicted in Fig. 11a,b, respectively. Figure 11a reveals that the maximum stress in the rib reconstruction model is concentrated near the junction of the artificial bone and the autologous rib, measuring 2.63 MPa. Similarly, the maximum deformation occurs near the junction of the artificial bone and the autologous rib, with a magnitude of 0.057 mm, as shown in Fig. 11b. Comparing the static analysis of a single rib with that of the overall rib reconstruction model, the stress distribution in the overall rib reconstruction model closely resembles that of a single rib. The primary difference lies in the significantly higher stress values at the rib junctions. For the reconstructed rib model, the maximum stress appears at the right side of the joint. Further analysis of the cause shows that since the rib is an asymmetric structure, the load is not uniform on both sides during the stressing process, so it appears on the right side. Also, since the joint connects different parts of the rib, it is subjected to higher loads here and hence the maximum stress occurs here.

Additionally, the overall rib reconstruction model exhibits reduced deformation. This phenomenon can be attributed to the overlapping sections of artificial and autologous bone, along with the connecting structure in the middle, which enhance the overall model's stiffness and effectively minimize deformation.

#### Dynamics analysis

The collision analysis for the current human bone and artificial rib reconstruction model shares the same configuration as the simulation setting for a single artificial rib. The simulation diagram and the trend in contact force change are depicted in Fig. 11c–e. As shown in Fig. 11e, it becomes apparent that the contact force change trend closely mimics that of a single artificial rib. The contact force experiences a rapid decline upon rib fracture, with a peak force of 574 N. In comparison to a single artificial rib, the force at the moment of fracture exhibits a notable improvement, indicating that the rib reconstruction model can withstand a more substantial impact force.

### Clinical application of artificial bone

**Figure 12:**
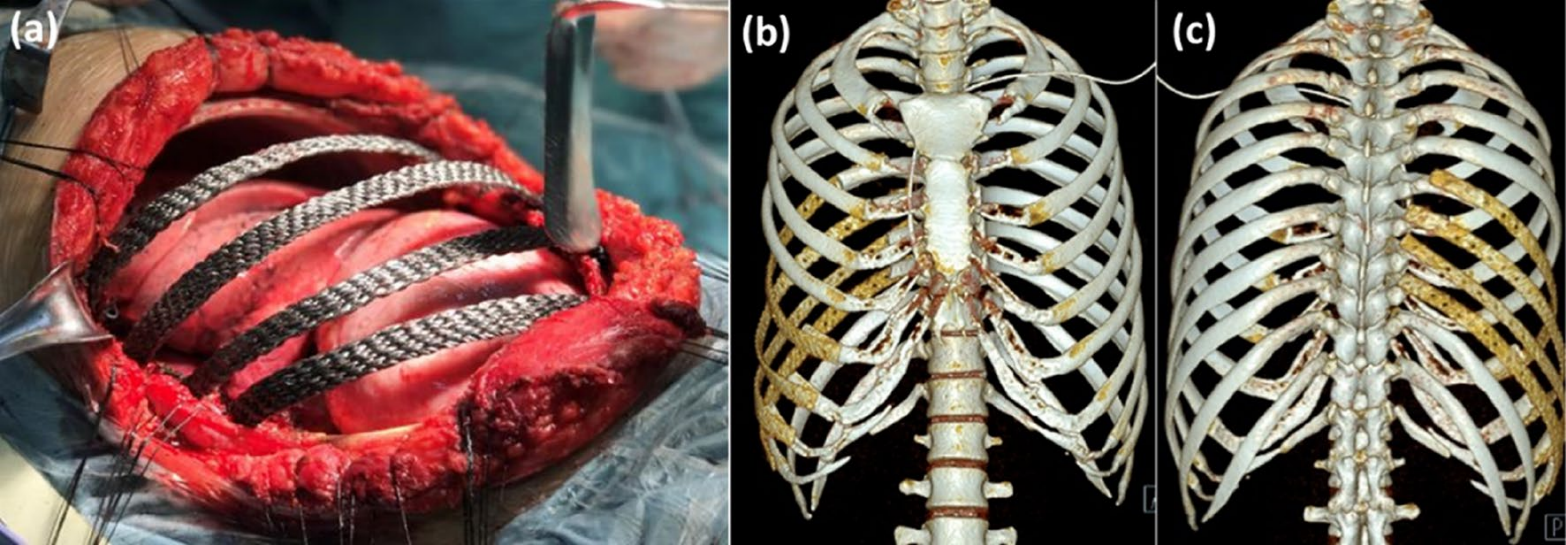
The right 6th to 9th ribs reconstruction at human implantation: (**a**) artificial rib implantation; postoperative CT image reconstruction, (**b**) anterior side; (**c**) rear side.

13 patients with chest wall defects were chosen for the chest wall reconstruction using carbon fiber artificial ribs. The detailed information is listed in Table3. Figure 12 shows a typical human implantation of carbon fiber artificial ribs on the right 6th to 9th ribs reconstruction. The postoperative CT image reconstruction shows the carbon fiber artificial ribs match well with the health human bones. According to the clinical research data on carbon fiber artificial ribs, 10 out of 13 patients showed no significant decrease in FEV1 (Forced Expiratory Volume in 1 s) and FEV1/FVC (Forced Expiratory Volume in 1 s to Forced Vital Capacity ratio) after surgery. This indicates that the implantation of carbon fiber artificial ribs effectively ensures normal breathing for patients.

**Table 3:** Clinical research data of carbon fiber artificial ribs.

No	Gender	Age	Implanta-tion site	Before implantation			After implantation		
				FEV1(L)	FEV1/FVC (%)	Function	FEV1(L)	FEV1/FVC (%)	Function
1	Male	40	Chest lock rib one piece	2.63	98.13	abnormal	2.19	82.0	Normal
2	Male	75	3rd to 6th ribs	2.18	73.5	Mild obstructive type	2.22	85.0	Normal
3	Female	47	4th rib	2.08	72.7	Mild obstructive type	2.33	81.4	Normal
4	Male	18	3rd to 5th ribs	2.63	85.0	Mild restrictive	2.45	89.2	Normal
5	Male	54	2nd to 3rd ribs	2.79	78.0	normal	2.47	78.0	Normal
6	Female	32	sternum	2.03	97.0	Mild restrictive	2.37	88.7	Normal
7	Female	46	4th to 6th ribs of sternum	2.03	75.6	Mild restrictive	2.23	83.1	Normal
8	Female	58	4th to 6th ribs of sternum	2.53	80.5	normal	2.36	84.7	Normal
9	Female	47	3rd and 4th ribs	2.04	78.0	Nonspecific changes	2.13	81.2	Normal
10	Male	53	2nd and 3rd ribs	2.53	82.0	normal	2.40	81.9	Normal
11	Female	29	6th to 9th ribs	2.31	84.6	normal	2.14	83.2	Normal
12	Female	61	sternum	1.57	81.0	Mild restrictive	1.69	85.3	Normal
13	Male	13	9th and 10th ribs	2.82	83.4	normal	2.89	84.8	Normal

## Conclusion

In this study, finite element analysis and clinical implantation were adopted to simulate and evaluate the biomechanics of carbon fiber artificial ribs under static and dynamic loading conditions like normal breathing and external impacts. It was found that the carbon fiber artificial ribs meet the mechanical requirements for implantation and maintain normal respiratory function as the stress on the artificial ribs under normal breathing is far below their strength limit. The whole artificial rib could withstand a frontal collision with a 4 kg rigid object at 2 m/s without damage. Reconstructing a single artificial rib with a human rib increased the overall stress and impact force required for fracture compared to a single artificial rib alone, suggesting improved strength and reliability. Clinically, 10 of 13 patients implanted with the artificial ribs showed no significant decrease in pulmonary function tests like FEV1, indicating normal breathing was maintained after implantation. As a result, the biomechanical simulations and clinical results demonstrate the carbon fiber artificial ribs have suitable strength and mechanics to serve as implants for chest wall reconstruction while maintaining normal respiratory function. Our study provides useful engineering validation of the artificial rib design and material prior to wider clinical use.

## Data Availability

All data generated or analysed during this study are included in this published article; further inquiries can be directed to the corresponding author.
